# Navigating the Gluten-Free Boom: The Dark Side of Gluten Free Diet

**DOI:** 10.3389/fped.2019.00414

**Published:** 2019-10-15

**Authors:** Aaron Lerner, Thomas O'Bryan, Torsten Matthias

**Affiliations:** ^1^Aesku.KIPP Institute, Wendelsheim, Germany; ^2^Gastroenterology, Clinical Practice and Institute of Functional Medicine, The National University of Health Sciences, San Diego, CA, United States

**Keywords:** celiac disease, gluten free diet, gluten withdrawal, side effects, nutritional deficiency, non-celiac wheat sensitivity, morbidity, mortality

## Abstract

In gluten dependent conditions the gluten free diet is the cornerstone of therapy, decreasing disease activity, improving health and quality of life and treating or preventing the associated complications. Gluten withdrawal implies strict and lifelong elimination not only of wheat, barley, rye, and wheat-contaminated oats, but also of numerous non-nutritional products where components of wheat are often added. Due to multiple reasons the diet is difficult to follow and the long-term adherence is decreased with time. The present review summarizes the dark side of gluten restriction where nutritional deficiencies, toxicity, morbidity, mortality, and mental health problems are reported. The aim being to increase awareness, avoid, detect and treat the side effects and to promote a healthier nutrition, for the patient's benefits.

## Introduction

The global population has more than doubled in the last 40 years supported by the “*green revolution”* in agriculture producing high-yield grain varieties, including semi-dwarf, high-yield, disease resistant varieties of wheat, that are central to the modern diet (1). Awarding the Nobel Peace Prize in 1970 to Norman Ernest Borlaug recognized the value of dwarf wheat to humanity.

Celiac disease has traditionally been clinically considered and then investigated with patients presenting with gastrointestinal (GI) symptoms. However, for every adverse reaction to wheat presenting with GI symptoms, there are 8 presenting without GI symptoms (2). Thus, dependence on GI complaints as a prerequisite in considering an adverse reaction to wheat, will allow a majority to escape diagnosis. This is a critical point of recognition for the Clinician when considering an association of wheat related disorders (WRD) and the potential value of a Gluten-Free Diet (GFD).

The 8:1 ratio of extra-intestinal vs. intestinal symptoms is not limited to celiac disease. In a prospective 1-year study of suspected non-celiac gluten sensitivity (NCGS) related disorders from 38 Italian centers—all recognized as referral centers of excellence and included in the register of the Italian Health Ministry for the diagnosis of gluten-related disorders, 53% of patients presented with non-abdominal complaints. The most frequent extraintestinal manifestations were fatigue and lack of well-being, reported by 64 and 68%, respectively, of the enrolled subjects. In addition, a high prevalence of neuropsychiatric symptoms including headache (54%), anxiety (39%), “*foggy mind”* (38%), and arm/leg numbness (32%) were recorded. Other extra intestinal manifestations emerging from the analysis of the survey responses were joint/muscle pain often misdiagnosed as fibromyalgia (31%), weight loss (25%), anemia (22%)—due both to iron deficiency and low folic acid, depression (18%), dermatitis (18%), and skin rash (29%), Umberto et al. (3). With its global impact in the body and lack of isolated tissue vulnerability, a high degree of suspicion is required for a Clinician to investigate a presenting patient for a WRD.

GFD, the mainstay of treatment for celiac disease (CD), is increasingly being adopted by people without a diagnosis of celiac disease. Gluten-free (GF) eating patterns have become a mainstream phenomenon during recent years, and nearly one-third of Americans report having attempted to eliminate or reduce the amount of dietary gluten they consume (4).

Currently, wheat is the most widely cultivated crop in the world and the dominant staple crop in temperate countries, providing between 20 and 50% of the total calories intake. The gluten family of proteins are the major proteins of wheat and are very important for wheat survival. In industrial countries, wheat makes substantial contribution to diet and health, particularly providing dietary fibers, B vitamins (B1, B2, B3, B6, B9) and mineral micronutrients, notably iron, zinc, and selenium. Hence, restricting wheat intake, without the guided support of a well-trained Registered Dietician, Nutritionist, or Physician can have serious consequences for the intake of essential nutrients and other beneficial components. GF menus are significantly lower in protein, magnesium, potassium, vitamin E, folate, and sodium, with suggestive trends toward lower calcium and higher fat (4). Equivalent sources of essential nutrients must be provided.

A common misnomer is that GF substitute foods are healthy. There is no evidence to support such a claim. While it is true that GF foods will eliminate a primary antigenic component of the diet, GF foods are calorie rich and nutrient poor. Restricting the intake of wheat in the diet can have serious consequences for the intake of essential nutrients and other beneficial components unless equivalent sources of these are provided (5). It is generally considered that GF foods are less nutritionally adequate than standard products. GF bread products were significantly higher in fat and fiber. All GF products were lower in protein than standard products. Only 5% of GF breads were fortified with all four mandatory fortification nutrients (calcium, iron, niacin, and thiamin), only 9% of GF bread products were fortified with thiamin, riboflavin, and niacin, and 28% of GF breads were fortified with calcium and iron only. This lack of fortification may increase the risk of micronutrient deficiency in celiac sufferers.

Based on the modern awareness on the nutrition-health bidirectional connections, the topic of healthy food is today's fashionista. According to the World Health Organization, a healthy food should prevent under and over nutrition and protect from chronic, non-communicable diseases (6). Controversially and in real facing of the mirror, the metabolic syndrome, allergy, cancer, stroke and autoimmune conditions are surging in the last decades (7–9). All 5 of these conditions have inflammation as the igniting spark associated with their development. Mirroring those alarming surges with simultaneous substantial declines in infectious diseases set the stage for the hygiene hypothesis. However, new scientific winds are blowing and “cleaning” the hygiene related concepts and prioritize the friendly microbiota over the pathogenic dysbiota in shaping gut homeostasis (10, 11). Changes in the microbiome on a GFD have substantial influence on the pro/dysbiotic environment of the GI Tract. It appears that intimate evolutionarily relationships are important, indispensable and crucial for bugs and us to develop and survive (12–14). We need them and they can't survive without our gut hospitality. Here comes the major environmental factor that keeps the physiological microbiome at bay: healthy, well-balanced food. Many food intolerances and allergies, metabolic conditions, genetic abnormalities, autoimmune diseases, and other medical conditions require special or controlled diets, GFD being one of them. Due to the incomplete adoption of a GFD outside the boundaries of a proven gluten related disease, the present review will concentrate on the dark side of gluten withdrawal.

### Gluten Dependent Conditions

Several diseases are considered to be gluten dependent. Gluten allergy that follows immune allergic mechanisms, celiac disease (CD), gluten ataxia and dermatitis herpetiformis as autoimmune conditions and the recently describe entity: non-celiac gluten/wheat sensitivity (15, 16). Celiac disease is a lifelong autoimmune disease affecting genetically predisposed individuals by the consumption of prolamins like wheat, barley, rye, and contaminated oats (17). It affects averagely 1–1.5% of Western populations, its incidence is surging worldwide even in far-east and under developed countries, a trend shared by many other autoimmune diseases (8, 18). Co-emergence of increased gluten consumption and CD prevalence and its recent changing epidemiology, reinforce the environmental over genetic influence in the contemporary CD surge. The only proven therapy is a lifelong GFD that reverse most of the symptoms, improve substantially mucosal pathology, near to the normal histology and prevent some of the long term complications. GFD can improve the other gluten dependent conditions. Prevent the allergy, alleviate the skin eruption in dermatitis herpetiformis, and potentially improve the ataxia in gluten ataxia. Despite being an effective therapy, multiple difficulties arise during its implementation.

### Gluten Free Diet

The only recommended and effective therapy for CD is strict exclusion of gluten for life. Despite strict adherence, complete mucosal recovery is rarely obtained. In most cases, a variable degree of inflammation persists characterized by persistent intraepithelial lymphocytosis with or without associated glandular hyperplasia (Marsh I-II/Grade A) at control biopsy usually performed after 6–18 months of GFD. Complete normalization to Marsh 0 stage was observed in 8% of patients, with Marsh I and II lesions persisting in 65% of patients with duodenal atrophy at baseline (19). And in the largest study ever done on Mortality and Celiac Disease, of 46,121 total biopsies, Marsh I-II was identified in 13,306 specimens. This was associated with a 72% increased risk of mortality in the first year after diagnosis (20). This is almost double the increased mortality in the first year after diagnosis with total villous atrophy CD (39%). The persistent duodenal intraepithelial lymphocytosis in CD patients during GFD is not eliminated by a gluten contamination elimination diet and is independent of the time length of GFD (21). GFD include withdrawal of wheat, barley, rye, contaminated oats, spelt, kamut, triticale, malt, or their hybridized strains. Natural gluten free nutrients like vegetables, legumes, fruits, fish or unprocessed meat, milk and dairy products, and eggs are allowed. By various industrial nutritional technics, gluten can be eliminated or decreased to <20 ppm, the amount allowed, for the CD patients by the European codex alimentarius (22). At least for now, GFD is fully recommended for CD, gluten allergy, dermatitis herpetiformis, and gluten ataxia. For the newly evolved entity of non-celiac gluten sensitivity, GFD might alleviate their symptoms, though, the pathophysiological pathways, offending environmental factors, strict diagnostic criteria and therapeutic nutrient withdrawal policy, are far from being resolved (23–25). The diagnostic criteria for CD and its related extra-intestinal manifestations (26, 27) are quite clear and the recommended gluten avoidance is obvious, but the lifelong restriction is a bumpy road (28, 29). The difficulties in following GFD and the reasons for poor patient's compliance were summarized recently (28, 29). Gluten is considered as a universal food additive as a thickener, flavor enhancer, filler, emulsifier, or texture modifier in many processed food products (6, 30). The present review will zoom on the deleterious effects of GFD, but, before will describe the nowadays fashionista of gluten/wheat avoidance.

## Gluten Withdrawal Fashionista

In parallel to the surge in gluten-related disorders prevalence, the numbers of people that empirically try to avoid gluten, for a variety of signs, reasons, and symptoms are gradually and continuously rising.

It becomes a very popular diet in contemporary history. Although many report improved health, increased energy, social and food industries and several best-selling books fuel its popularity, there is little evidence to explain the above mentioned reports. The opponents to and the approvers of gluten came to the forefront of popular nutritional discussions and “going gluten-free” has become mainstream in Western populations (31, 32). Currently, ~25% of the population reports keeping a GFD, many without adequate knowledge “requesting GF meals and ordering bread pudding for dessert” (33, 34). On the other hand adherence to GFD by the gluten dependent patients is poor and deserve continuous nutritional education and behavioral reinforcement programs (35, 36). No doubt, gluten restriction became today's fashion trend (37).

Recognizing that dietary changes alter the gut microbiome in humans within 24–48 h (38), a 2011 study demonstrated that for celiac children following a GFD for 2 years, the GFD did not completely restore the microbiota and, consequently, the metabolome of CD children (39). In another study, 10 healthy participants who did not have CD or a recognized gluten sensitivity, following a GFD, found that their diet alone created a pro-inflammatory GI environment 100% of the time. Researchers concluded that when individuals discontinue eating wheat, the reduced polysaccharide intake (prebiotics) creates a shift in the diversity of the intestinal microbiome, with lowered *bifidobacterium* and *lactobacillus* and a concomitant increase in *enterobacteriaceae*, diminishing short chain fatty and organic acids and thus reducing competence of the immunomodulatory role of the microbiome with lowered TNFα, IFN gamma, IL-8, and IL-10. This outcome creates a pro-inflammatory environment (40).

With wheat providing 78%, and barley (3%), together providing 81% of oligo fructose and inulin for average North Americans (41), a non-guided GFD, which does not purposefully replace these critical fructan prebiotics (such as Arabinoxylose), runs the risk of creating a starvation state for the resident probiotics, easily creating an inflammatory cascade environment in the microbiome. Without these fructan prebiotics in wheat, the microbiota which was accustomed to this family of prebiotics die off, creating a pro-inflammatory environment (42).

An example of the potential complication of the above dynamic is the production of trimethylamine N-oxide. This metabolite of choline increases atherosclerotic plaque size, triggers prothrombotic platelet function, promotes arterial thrombus growth (43), and predicts risk for cardiovascular diseases (44). This is produced by action of genera *Allobaculum* and *Candidatus arthromitus* as well as the family *Lachnospiraceae, which* were identified in the cecal microbiota producing higher levels of serum trimethylamine N-oxide (45). Hence, the composition of the commensal microbiota is an emerging risk factor for cardiovascular diseases.

In view of the rising popularity of gluten restricted products, the present review will summarize the dark side of the GFD, rather than describing the macro and micronutrients deficiencies, extensively described in naïve or untreated CD patients (46).

## Nutritional Deficiencies

GFD is an unbalanced diet that buries in its lap multiple nutritional deficiencies. It is known that gluten restricted products are of poorer nutritional value, lower quality, unpleasant mouth-feel, less flavored and diminished viscoelasticity of bread products. Table 1 summarizes those deficiencies while being treated with GFD. The fact that most of the GFD products are not fortified, further aggravates the deficiencies. Summarizing surveys on nutritional profiles of gluten free, compared to gluten containing food products, Melini et al. showed that the GFD is deficient in fibers, proteins, folate, iron, potassium, and zinc, while higher in fat, carbohydrate, sugars, FODMAP's, and sodium, in most of the surveys (6).

**Table 1:** Nutritional deficiencies, excesses and toxicity in gluten free products.

**Nutrient abnormality/deficiency**	**Excessed components**	**Toxicity**
Iron	Proteins	Heavy metals: arsenic, mercury, lead, cadmium
Calcium, sodium	Fat, saturated fatty acids	Food additives: enzymes like microbial transglutaminase, proteases
Vitamin D	Sugar, sucrose	
Vitamin C	Energy intake	
Vitamin A, E		
B Vitamins: B12, thiamin, riboflavin, niacin	Fat, saturated fatty acids	
Folate	High Omega 6 fatty acids	
Zinc, Magnesium, Selenium	Higher arachidonic acid: docosahexaenoic acid ratio	
Fibers: oligo fructose, inulin, fructans	Higher pro-inflammatory fatty acid profile	
Low HDL, Apo A1		
Lower essential amino acids		
Low arachidonic acid: dihomo-γ-linolenic acid ratio		

*Adapted from Taetzsch et al. (4), Melini and Melini (6), Lerner et al. (13), Lerner and Matthias (30), Dennis et al. (46), Penagini et al. (47), Shepherd and Gibson (48), Theethira and Dennis (49), Hosseini et al. (50), Freeman (51), Lerner and Matthias (52), Matthias et al. (53), Matthias and Lerner (54), Lerner and Matthias (55), Allen and Orfila (56), Riezzo et al. (57), Lamacchia et al. (58), and van Hees et al. (59)*.

The substitution of gluten containing prolamins by rice and corn put the patient at risk of protein, fiber and folate deficiencies (50, 60) and higher glycemic index (60). Thus, partially explaining the higher rate of metabolic syndrome and cardiovascular morbidity reported in naïve and treated CD patients (61–64). One must remember the association of an activated immune response to other foods in over 50% of celiacs maintaining an inflammatory environment in the gut (65).

## Toxicity

There are two main sources for toxic or potentially deleterious compounds, while consuming GFD. The first one is due to the common consumption of fish and rice. Both have increased levels of heavy metals like lead, cadmium, mercury, and arsenic [(51), Table 1]. In a recent study, persons following GFD, including non-celiac patients, had increased urinary levels of arsenic and blood levels of mercury, lead, and cadmium compared to persons consuming gluten (66). Since most of the GFD consumers today are non-celiac people, those results relay the accumulation of the toxic heavy metals to the gluten restrictive diet, rather than to CD (67). An additional reason to avoid the gluten-free “fashionistic” fad.

The second potential source of toxic harmful compounds is the enzymes used as food additives in the processed food gluten free products [(50), Table 1]. Microbial transglutaminase (mTg) imitates functionally the autoantigen of CD, namely, the tissue transglutaminase (52). It is a very common additive for cross linking proteins, thus, changing the physical properties, 3D structure, and immunogenicity by exposing new epitopes, and food quality of many GF products, including in the bakeries (50, 52, 53). Most recently, the enzyme was suggested as an environmental factor that drive CD development (52–55). Its potential deleterious effects in GFD, deserve further exploration.

## Increased Morbidity and Mortality

Undiagnosed or untreated CD patients are at risk for acute (68) or chronic morbid manifestations and associated diseases. However, when compliant to GFD the risk for morbidity and early mortality continues. The background might be inherent to GFD content or to their continued intestinal inflammation. Table 2 summarizes the morbid conditions that CD population on gluten restricted diet might risk.

**Table 2:** Summary of the causes of increased morbidity on GFD.

**Increased morbidity**	**References**
Social leisure activities	(32, 50)
Metabolic syndrome	(64)
Hepatic steatosis	(64)
Cardiovascular risks	(4, 62, 63)
Depression	(56)
Diabetes 2	(4)
Osteopenia/ osteoporosis	(69)
Reduced beneficial microbiota	(42, 70)
Loss of around 80% of prebiotics	(42)
Obesity	(4, 71)

The increased mortality in CD populations was previously reported. In the largest survey of mortality in CD, mortality was increased in CD, in latent CD and in patients with intestinal inflammation, hazard ratio (HR) 1.39, 1.35, and 1.72, respectively (20).

The HRs for mortality were highest in the first year of follow-up after diagnosis with total villous atrophy CD associated with a 2.80-fold increased risk of death, latent (positive serology, negative histology) celiac disease with a 1.81-fold, and inflammation (increased intraepithelial lymphocytes) with a 4.66-fold increase. These startling statistics should bring pause to every Clinician. The only difference post diagnosis for the vast majority of newly diagnosed celiacs is implementation of the GFD. With maintaining inflammation in the intestines on a GFD, the HR for mortality in the first year post-diagnosis was almost double that of total villous atrophy CD. The necessity of comprehensive education for transitioning the patient to a healthy GFD, free from emphasis on GF commercial foods high in calories and low in nutrients, cannot be overemphasized. The most common cause of death in the first year post diagnosis was cardiovascular diseases, followed by malignancy. Excess mortality was independent of the intestinal damage and was observed even on GFD, most probably due to the persistent mucosal inflammation. In fact, after an average of 12 years on a GFD, 31% of patients still have increased enteric inflammation (72). Suicide rate is increased in CD, latent CD and persistent intestinal inflammation with HR of 1.55, 1.06, 1.96, respectively, compared to the general population, when done on a Swedish cohort (73). Interestingly but alarming, CD diagnosed in childhood was associated with a 40% increase in suicide risk. It should be noticed that naïve and treated CD is frequently comorbid with multiple additional autoimmune diseases like autoimmune thyroiditis, type 1 diabetes, IBD and many more (14, 26, 27, 74). Cancers like non-Hodgkin lymphoma and other gastrointestinal malignancies and genetic conditions like Downs, Turner, and Williams syndromes are also associated with CD, thus adding to the morbidity and mortality, including when on gluten withdrawal. At the end of the day, it is possible that persistent inflammation increased both overall mortality rate and suicide risk, despite following GFD.

## GFD and Mental Health

Evidence points to the nervous system as the prime site of gluten damage. Literature reported that gluten can cause neurological harm through a combination of cross reacting antibodies, immune complex disease, and direct toxicity (75). The association between CD and other gluten related conditions with behavioral and psychiatric disorders was extensively reported and summarized recently (76–78). The spectrum includes anxiety, dysthymia, depression, attention-deficit hyperactive disorders, mood and sleep disorders, mal social adaptation, substance-related, addictive and neurocognitive disorders, suicide attempts, learning disabilities, feeding and eating disorders, bipolar syndromes, schizophrenia, and autistic spectrum disorders. Those disorders appeared across different age groups, in both genders and some are considered as extra-intestinal manifestation of CD. The strongest association was reported with depression (59, 76), but, the association between chronic medical conditions and depression is well-established. It might be bidirectional since physical illness or disability often worsen the mental disorder and vice versa (79). More so, dietary modification, including GFD in gluten related conditions, may be associated with pathological eating practices (80). Compliance with GFD is a tough alley (28), associated with major changes in daily lifestyle routine and activities and eating habits, many times stressful and difficult to accept (81, 82). The underlying mechanisms that induce those psycho-social-behavioral disorders, while on GFD, are not understood. Nutritional and vitamin deficiencies (59), an immune dysregulation, cerebral hypo perfusion (83), the gut “feeling of the brain” in the gut-brain axis (84) or the stress of adherence to GFD, have been suggested (76).

## GFD Side Effects: Closing the Gaps

Taking into account the tough alley and the contemporary torrid times in complying with a strict GFD (28), sincere and practical efforts should be implemented to close the gaps. Following are some suggestions to avoid the detrimental effect of GF product consumption toward a more personalized nutrition, tailored to the individual needs.

Gluten restriction should be accompanied by healthier food to avoid nutritional deficiencies, toxicity of alternative non-gluten containing prolamins, morbidity and finally early mortality. In this regards, it is advised to adapt the Mediterranean diet, the most studied and successful one against major non-communicable chronic diseases (85). It is rich in fibers, anti-oxidants, anti-inflammatory mediators, vitamins, trace elements, minerals, beneficial fatty acids, qualified proteins, bone building compounds, metabolically balanced and affects the enteric microbiome favorably (86, 87). Enriched cow's milk and dairy products are salutogenic to CD patients and their lactose intolerance is transitory while on gluten restriction (88, 89). If a patient has neither clinical nor serology evidence of a sensitivity to cow's milk (90), and they are not homozygous for a primary lactase deficiency (91), the nutrient dense platform of milk may be of substantial value in supplying macro and micro nutrients for rebuilding an inflammatory-damaged terrain.

Asking a patient to abstain from a most-common food in their diet, often eaten in some form multiple times per day, is an extremely challenging directive fraught with non-compliance absent expert on-going advice. Early and effective consultation with a gluten knowledgeable, food labeling skilled, Dietitian, Nutritionist or Health Coach (28), increasing the intake of pseudo-cereals, polyphenol-rich fruits and vegetables, encouraging natural GF foods, focused efforts on rebuilding a balanced non-inflammatory microbiome, increasing the patient's education on the disease evolvement, GF products and follow ups, in order to improve adherence and establish a healthy balanced diet (6, 47, 49, 50, 92) is of essential nature in reducing the increased risk of morbidity and mortality. Very important is the physician-dietitian-patient trustful relationship and the team communication for the patient's successful recovery and adherence to the dietary restriction. Not less important is the periodic laboratory test to check for CD associated serological markers and to detect any nutritional deficiencies.

## Conclusions

GFD is the only proved therapy for CD and is crucial in the other gluten related conditions. It reduces activating an inflammatory cascade in the intestines, rehabilitates the intestine, normalizes the associated biomarkers and can treat or improve some of the extra-intestinal manifestations. However, despite being an effective therapy, GF products suffer from limitations, side effects and might bear risks if not detected and dealt appropriately. Nutritional deficiencies, toxic components, unbalanced diet, increased morbidity, mortality and psychiatric or behavioral abnormality, are a few examples. The treating team should be aware of the potential dark side of gluten restriction, diagnose those abnormalities and offer adequate solutions to improve the patient's health and quality of life.

## Author Contributions

AL and TO’B: conceptualization. TO’B: methodology. AL: formal analysis. TM: resources. AL and TO’B: data curation. AL: writing—original draft preparation. AL, TO’B, and TM: writing—review and editing. All authors read and approved the final manuscript.

### Conflict of Interest

The authors declare that the research was conducted in the absence of any commercial or financial relationships that could be construed as a potential conflict of interest.

## Abbreviations

CD: celiac disease
GF: gluten free
GFD: gluten free diet
GI: gastrointestinal
NCGS: non-celiac gluten sensitivity
WRD: wheat related disorders.
